# Nurses’ attitude towards patient advocacy in a single tertiary care hospital

**DOI:** 10.1002/nop2.958

**Published:** 2021-06-25

**Authors:** Fahad Zeed Alanezi

**Affiliations:** ^1^ King Fahad Medical City Riyadh Saudi Arabia

**Keywords:** nurses, nursing, patient advocacy

## Abstract

**Aim:**

To determine nurses’ attitude towards patient advocacy in a single tertiary care hospital.

**Design:**

Descriptive/analytical cross‐sectional studies.

**Methods:**

A comprehensive two‐part questionnaire about nurses’ views on nursing advocacy was administered to 371 nurses using a convenient random sampling. The first part included eight demographic variables, and the second part, the questionnaire, was used to measure nurses’ attitude towards patient advocacy.

**Results:**

Nurses were more likely to act as patient advocate when their patient was in danger, and their employment was not at risk while acting as patient advocates. Female nurses scored higher than males; those with higher qualifications had higher behavioural and cognitive scores. A significant correlation was observed between cognitive (belief) aspects of attitude (*p* = .78, *p* ≤ .001) and behavioural (efficacy) aspects (*p* = .89, *p* ≤ .001).

## 1 INTRODUCTION

Nurses serve as patients’ guardians, social workers and persons who do everything in hospitals for the benefit of their patients. When patients are in jeopardy, nurses act as their advocate. Patient advocacy is an essential part of the nursing profession (Matthews, 2012). Throughout our literature search, patient advocacy is perceived as a fundamental nursing component. Every day, nurses face many challenging situations. Each of these requires an intervention to maintain patient safety (American Nurses Association (ANA), 2019; Canadian Nurses Association (2019)). Merriam Webster (2017) defines advocate as “one who pleads the cause of another; *specifically,* one who pleads the cause of another before a tribunal or judicial court.” “Patient advocacy is a pillar of nursing, making it unique from all other professions” (Graham, 2012). Nurses can protect their vulnerable patients from any unsafe actions of incompetent healthcare professionals and maintain their patients’ safety (Davoodvand et al., 2016). According to the ANA (2017), “dedication to patient safety and nursing quality comes in the form of advocacy.” By creating initiatives that raise awareness both among legislators and the general public, ANA can encourage the legislation on important issues such as safe patient handling and patients’ rights.

## 2 BACKGROUND

According to Mongan, Long and Farragher (2016), the most common six models in the literature are as follows:

**“Self‐advocacy”** individuals are empowered to speak up.
**“Group advocacy”** individuals shared their experiences in groups who speak up collectively about issues.
**“Peer advocacy”** involves “one‐on‐one” service where they share their experiences to support each other express and match their needs.
**“Citizen advocacy”** citizens are empowered to be involved with a person who may need support.
**“Independent/representative advocacy”** trained advocates are hired to deal with special issues and work with an individual until that issue is solved.
**“Legal advocacy”** is intended to protect the rights of people through a legal system.

For nurses to be patient advocates, there will be undeniable consequences that benefit the relationship between nurses and patients; however, it does expose nurses to many crucial challenges and barriers (Kupperschmidt, 2014). Some barriers deterred patient advocacy; with regard to education, nurses required more advocacy educational programmes and support from their employers if they were to play such a role (Mortell et al., 2017). This could be achieved by extending the nursing trainee curriculum and through better quality nursing advocacy plans (Mortell et al., 2017). The second barrier was the way nurses show empathetic care for their clients (Josse‐Eklund et al., 2014). In addition, several organizational barriers do not have the system and its policies to support nurses (Josse‐Eklund et al., 2014; Mortell et al., 2017). For instance, some nurses reported that they were scared because they believed that the organization did not secure them if they acted as a patient advocate. Therefore, some nurses feel insecure when they have issues with physicians as doctors wield the power in such organizations. Moreover, nurses believed that to effectively function as a patient advocate, a policy guiding and protecting them from other professions should be clear (Mortell et al., 2017). However, a study that recruited only Saudi nurses that made it difficult to generalize its outcomes. In addition, another study conducted by MORTELL (2018) recruited only Saudi nurses and found that the perceptive of the Saudi nurses towards patient advocacy remains controversial because they believed that issues associated with their role as patient advocates are an optional practice. Several studies conducted in Saudi Arabia were in small groups; therefore, their conclusion cannot be generalized. Moreover, another study recruited Muslim nurses alone and could not generalize their outcomes (Mortell et al., 2018). Therefore, this study was conducted to shed more light on this research area, this study.

### 2.1 Research question

What are the nurses’ attitudes towards patient advocacy in a single tertiary care hospital?

## 3 THE STUDY

### 3.1 Design

Descriptive/analytical cross‐sectional studies.

### 3.2 Method

This study was conducted in King Fahad Medical City (KFMC) in Saudi Arabia. The study population included all male and female nurses who worked in emergency departments, intensive care units, medical and surgical wards, outpatients, children wards, maternity wards, oncology wards, cardiac wards, neurosurgery wards and rehabilitation wards. Different departments at King Fahad Medical City (KFMC) were included. The sample was conducted by the sample size calculator of prevalence studies by considering 50% proportion patient advocacy among nurses with 95% confidence interval and 5% margin of error. Hence, a total of 371 nurses were included in this study.

Out of the total 2,650 nurses working at KFMC, a questionnaire was administered on 371 nurses on convenient random sampling. A comprehensive two‐part validated questionnaire was used to gather data for nurses’ view towards nursing advocacy. Requisite permission was obtained from Motamed‐Jahromi et al. (2013) before conducting this study.

The first part included seven demographic variables: hospital name, age, gender, marital status, work experience, educational background and previous participation in patient advocacy workshop. The second part consisted of questions pertaining to attitude measuring instruments for the nursing advocacy and each question score ranged from one–five using a five‐point Likert scale: (i) strongly disagree, (ii) disagree, (iii) neither, (iv) agree and (v) strongly agree. Nine of these questions were formulated positively, and ten were stated in a negative way. English was the official language in KFMC, and since the study population consisted of nurses at KFMC, the questionnaire was in English and all participants were able to understand the questions. The questionnaires were originally written in English, and the cross‐cultural adaptation was also carried out. These questionnaires were distributed as hard copy.

A factor analysis of the questionnaire was carried out by Motamed‐Jahromi et al., (2013). Their assessment revealed that data were factorable using a Kaiser–Meyer–Olkin (KMO) test of sampling adequacy, 0.85, and Bartlett's test of sphericity, *p*‐value = .00 for factor analysis. They performed factor analysis using the principal component analysis (PCA) varimax rotation scheme. By examining the eigenvalues and using the scree plot, they identified two factors: factor one for cognitive (believe) aspect of attitude (10 items) and factor two for behaviour (efficacy) aspect of attitude (nine items).

### 3.3 Analysis

Demographic characteristics of study participants were reported as counts (percentage). To examine the difference in attitude, mean score and demographic variables, one‐way analysis of variance (ANOVA), Kruskal–Wallis test and Student's *t* test were performed, as appropriate. Spearman's correlation was used to evaluate the correlation between the two nurse attitude factors and age. All statistical analyses were performed using SPSS 25.0 software (SPSS Inc., Chicago, IL, USA) package. Based on a two‐tailed test, a p‐value of 0.05 was considered significant.

### 3.4 Ethics

The Research Ethics Committee approval for the study was obtained by the institutional review board. All participants consented before participating in the study; no known risks were associated with this research and study purpose. Data anonymity and confidentiality were maintained throughout the study. Questionnaires were distributed by the primary investigator to each participant and later collected by the primary investigator and kept in a private place where no one can access them, and their names were not written in the questionnaire. The STROBE checklist was also used in the study.

## 4 Results

Table 1 describes the demographic data. Participants aged 22–53 years (mean, 34.59 years and standard deviation 6.43), female nurses, 66.3% were married and 32.1% were single. About 69.5% of participants had a bachelor's degree in nursing, followed by 26.4% those who had a diploma degree. Only one participant had a PhD, and only 4.0% had a master's degree. The work experience ranged from 1–>20 years. About 35.3% nurses had a work experience from 6–10 years, whereas 35.3% had work experience of >20 years and 25.9% of the staff had an experience from 11–15 years. Participants’ work areas were from different centres and hospitals at KFMC as shown in Table 1. Only 17% of participants had ever attended patients’ right workshop.

**TABLE 1:** Participants’ demographic characteristics

		*N*	%	Mean	Standard Deviation	*p*‐value
Age category	<30	80	21.6%	4.12	0.49	.08
	30–40	227	61.2%	4.00	0.50	
	>40	64	17.3%	3.93	0.67	
Gender	Male	36	9.7%	3.79	0.73	.06
	Female	335	90.3%	4.03	0.50	
Marital status	Single	119	32.1%	4.09	0.51	.05
	Married	246	66.3%	3.96	0.54	
	Widowed	5	1.3%	4.23	0.69	
	Divorced	1	0.3%	4.84		
Hospital	Main hospital	84	22.6%	4.02	0.45	.05
	KSCH	52	14.0%	3.86	0.61	
	CCC	52	14.0%	4.13	0.52	
	NNI	25	6.7%	4.07	0.49	
	OPD	35	9.4%	3.77	0.82	
	Rehab	38	10.2%	4.02	0.47	
	WSH	50	13.5%	4.13	0.44	
	CSH	35	9.4%	4.07	0.35	
Educational level	Diploma	98	26.4%	3.90	0.49	0.034
	Bachelor	258	69.5%	4.06	0.53	
	Master and above	15	4.0%	3.93	0.47	
Work experience	1 = 1–5 years	76	20.5%	4.06	0.48	.38
	2 = 6–10 years	131	35.3%	4.05	0.56	
	3 = 11–15 years	96	25.9%	3.99	0.48	
	4 = 16–20 years	49	13.2%	3.94	0.42	
	5 = more than 20 years	19	5.1%	3.79	0.89	
Participation	Yes	63	17.0%	4.08	0.48	.23
	No	308	83.0%	4.00	0.54	

Abbreviations: CCC, Comprehensive Cancer Center; CSH, Children's Specialized Hospital; KSC, King Salman Heart Center; NNI, National Neurosciences; OPS, Outpatient Department; *p* = level of significance; Rehab, Rehabilitation Hospital; WSH, Women's Specialized Hospital.

Comparing the mean scores of participants’ perception towards patient advocacy and their demographic characteristics revealed a difference between the mean score between male and female nurses. The mean score for male nurses was 3.75 out of five, whereas female nurses’ score was 4.03 with *p* = .06. Participants’ score age categories (*p* = .08), marital status (*p* = .05), nursing work experience (*p* = .38), educational level (*p* = .034), hospitals (*p* = .05) and participants at different age participating and non‐participating in Patient Right’s Workshop.

### 4.1 Descriptive findings

The descriptive analysis in Table 2 shows a positive attitude from participants with the mean score overall 4. Most nurses who participated in the study stated that they were good patient advocates due to their commitment on their job (mean = 4.52). In addition, most participants stated that they act as patient advocate when their patients asked them to (mean = 4.09). However, nurses stated fairly that a positive score towards patient advocacy was not a part of their job (mean = 3.45). Nevertheless, participants positively agreed that they acted as patient advocate to preserve patient's dignity mean = (4.44). Moreover, participants disagreed that their employment was at risk if they act as patient advocates (mean = 3.72).

**TABLE 2:** Mean score in questionnaires (the item range = 1–5)

Factor 1 = cognitive(belief) aspect of attitude	Mean	Standard Deviation
Q1 I am a good patient advocate because I’m committed to my job	4.52	0.62
Q2 I am ethically obligated to act as the patient advocate when patients are perceived to be in danger	4.50	0.67
Q3 I protect the patient's decision‐making right with nursing advocacy	4.42	0.68
Q4 I believe that nursing advocacy is not a part of nurses’ professional duties*	3.45	1.37
Q5 I certainly act as a patient advocate when the patient asks me	4.09	0.90
Q6 I act on patient's behalf and patient's speaker	4.01	0.88
Q7 The nurse and patient can simultaneously act as a patient advocate	4.14	0.73
Q8 Nurses act as patients’ representatives	4.07	0.84
Q9 Nurses protect patients’ rights in the healthcare environment	4.51	0.66
Q10 I act as a nurse advocate to preserve the patient's dignity	4.44	0.68
Factor1_avg	4.22	0.51

Factor 2 = behaviour(efficacy) aspect of attitude	Mean	Standard Deviation
Q11 I doubt my own ability to provide advocacy for patients. *	3.73	0.93
Q12 My employment is at risk when I act as a patient advocate. *	3.72	0.96
Q13 I face retribution from employers when acting as a patient advocate.*	3.55	0.94
Q14 My employer is not satisfied with me when I inform my patients of their own rights.*	3.88	0.91
Q15 Patient advocacy is why I get labeled as disruptive.*	3.73	0.91
Q16 I think nursing advocacy is not unconditional protection of patient.*	3.70	0.98
Q17 I did not play an effective role in nursing advocacy so far.*	3.85	0.93
Q18 I cannot protect patients from harmful situations.*	4.02	0.92
Q19 I do not have enough time for patient advocacy.*	3.87	0.96
Factor2_avg	3.78	0.77

Note

Negative statement = *

### 4.2 Correlation analysis

A significant correlation was observed between cognitive (belief) aspects of attitude (*p* = .78, *p* ≤ .001) and behavioural (efficacy) aspects (*p* = .89, *p* ≤ .001) (Table 3). However, there was no correlation between age of the participants and nurses’ attitude towards nursing advocacy. The mean score of staff who worked for >20 years is 3.79, whereas those who had 1‐ to 10‐year experience scored a mean of 4.05. Regarding the educational level, a significant positive correlation was observed among the staff who had higher education with higher score (*p* = .034).

**TABLE 3:** Correlation between mean of nurses’ attitudes with aspects of attitude and age of the participants

Scales		Correlation coefficient	*p*‐value
Attitude subscales	Factor 1 = cognitive(belief) aspect of attitude	*ρ* = 0.78	<.001
	Factor 2 = behaviour(efficacy) aspect of attitude	*ρ* = 0.89	<.001
Demographic factor	Age	*ρ* = −0.09	.09

## 5 DISCUSSION

A significant correlation was found between cognitive and behavioural aspect towards nurses’ attitude when it comes to patient advocacy. Nurses who showed positive attitude were most likely to act as patients’ advocate as compared with those who did not possess a higher attitude and behavioural aspects. As concluded by Nsiah et al. (2019), having a positive attitude towards patient advocacy made the nurses to fight more for the patients’ right when they had to. With regard to the correlation between the staff experience and positive attitude towards patient advocacy, the study did not find a positive correlation. However, the correlation was negative, but not statistically significant.

With this information, a study conducted by Thacker (2008) concluded that there was no association between work experience and positive attitude towards patient advocacy. Most participants agreed that both nurses and patients can simultaneously act as advocates for patients. The relationship between nurses and patient is an essential part of patient advocacy, and therefore, Manasco, McGoldrick, Kajana, Rosenthal, McMikel and Lins (2015) developed a framework for patient safety. Patients believe that it is their right to know about their condition and to advocate for their lives when they are in a vulnerable situation. Consequently, they need an external support from nurses to be their voice and nurses are committed to providing patient advocacy (Davoodvand et al., 2016). Most participants reported that they were ethically obliged to be patient advocates and could also advocate for their patient when they were in danger. Nurses are committed to advocating for their patients because it is in their moral compass as the first level of patient advocacy influencers (Josse‐Eklund et al., 2014; McGrath &). When it comes to positive attitude about patient's advocacy, no correlation was observed between nurses who attended patient's advocacy workshops compared with those who did not. However, according to the study done by Barrett‐Sheridan (2009) highlighting that education programmes for patient advocacy were correlated with having a positive attitude towards patient advocacy. As a result of that, educational programmes are recommended to improve the awareness of the importance of patient advocacy as the participants scored fairly a positive score towards that a patient advocacy is not a part of their job (mean = 3.45). In addition, a qualitative study conducted by Nsiah et al. (2019) reported that education was an essential part for patient advocacy.

One of the main dilemmas that faces patient advocacy is the organizational support. In this study, nurses stated that they face retribution from employers when acting as a patient advocate with score of 3.55 out of five. Many qualitative studies revealed that employees did not believe that they were supported from their organization, labelled as disruptive or their job might be at risk (Josse‐Eklund et al., 2014; Mortell et al., 2017). In contrast, in this study, 74% of nurses reported that their job was not at risk when they acted as patient advocate.

### 5.1 Limitations

The study was conducted in only one tertiary care centre in Riyadh, and the results cannot be generalized. It seemed that adopting a mixed methodology would strengthen the study's results and increase its generalization.

## 6 CONCLUSION

Patient advocacy should be maintained to have quality patient advocacy. Most nurses in the study showed a positive attitude towards patient advocacy. Scores varied when it came to work experience and gender. Qualification level among nurses was associated with having higher behavioural and cognitive scores. Educational programmes and reinforcement for the importance of patient advocacy for nurses are important and should be carried out more frequently. Further qualitative studies are recommended to measure the barriers towards patient advocacy and to explore their experiences.

## CONFLICT OF INTEREST

There is no conflict of interest.

## Data Availability

The data that support the findings of this study are available on request from the corresponding author. The data are not publicly available due to privacy and ethical considerations.
